# 810 A Bidirectional Translational Approach to Evaluate Perioperative Fluorescence Imaging for Burns

**DOI:** 10.1093/jbcr/iraf019.341

**Published:** 2025-04-01

**Authors:** Mary Junak, Hector Garcia, Aiping Liu, Bailey Donahue, Joana Pashaj, Emily Klossowski, Adam Uselmann, Lee Faucher, Brian Pogue, Angela Gibson

**Affiliations:** University of Wisconsin Madison; University of Wisconsin Madison; University of Wisconsin Madison; University of Wisconsin Madison; University of Wisconsin Madison; University of Wisconsin Madison; OnLume Inc.; University of Wisconsin Madison; University of Wisconsin Madison; University of Wisconsin School of Medicine and Public Health

## Abstract

**Introduction:**

The primary method to evaluate healing capacity of a burn wound is visual assessment; a subjective interpretation that risks over-excision. This ongoing study investigates the use of indocyanine green (ICG) fluorescence imaging to evaluate burn depth and healing potential. Early enrollment challenges encountered with the heterogeneity of human burn wounds, difficulty of patient recruitment early after a trauma, and variable timing of assessment post-injury led to the addition of a parallel translational pre-clinical swine model.

**Methods:**

Human subjects (n = 13) and adult pigs (n = 2) with burns of various depths received a 7 mg injection of ICG for angiography (ICGA) followed by a 5mg/kg intravenous (IV) infusion of ICG on post-burn day (PBD) 2 or 3. Second window indocyanine green (SWIG), a novel method of delayed fluorescence imaging, was performed the day after ICG injection on a region of interest (ROI) during wound care. A full thickness skin biopsy was taken from the center of the ROI and ICG microscopy and histologic staining was performed for tissue architecture and viability. MATLAB R2023b was used for data processing and GraphPad Prism 8.0 was used for statistical analyses.

**Results:**

In patient samples, there was high interpatient variability between histologic cellular viability and perfusion as measured by ICGA peak fluorescence intensity when looking at the ROI (R2 = 0.04). Microscopically, the tissue exhibited intense inflammatory cell infiltrate at the interface of viable and nonviable tissue in some tissues without correlation to need for skin grafting. Patients had a wide range of SWIG fluorescence intensities and patterns. Qualitatively within patients, ICGA and SWIG signals exhibit inverse fluorescence intensities. In our pig model, there was a strong linear correlation between increasing burn depth and decreasing ICGA peak fluorescence intensity (R2 = 0.84). Additionally, samples with a burn depth < 50% demonstrated significantly higher SWIG fluorescence intensity compared to those > 50% (p = 0.003).

**Conclusions:**

Variability in perfusion and inflammation contributes to heterogeneity in the ICGA and SWIG parameters in patients. The swine model supports our hypothesis that ICG signal is dependent on inflammation, permeability and destruction of the vessels affecting ICG delivery to the wound. However, the animal model simplifies the true complexity of the burn microenvironment and may contribute to clinical failure during translation to human subjects.

**Applicability of Research to Practice:**

The experimental approach we have pivoted to given the known challenges with human subjects research, exacerbated in trauma and burn injury, represents a paradigm shift. This iterative, bidirectional approach using human subjects and animal models in parallel, allows more nimble experimental design modifications and data interpretation to ensure translational success.

**Funding for the Study:**

NIGMS 5R01GM145723

